# The Association Between Psychosocial Stress and Perinatal Maternal Depressive Symptoms: A Case–Control Study in a Regional Medical Center in Hungary

**DOI:** 10.3390/jpm15070287

**Published:** 2025-07-03

**Authors:** Anita Sisák, Evelin Polanek, Regina Molnár, Andrea Szabó, Ferenc Rárosi, Armita Hosseini, Gábor Németh, Hajnalka Orvos, Edit Paulik

**Affiliations:** 1 Department of Public Health, Albert Szent-Györgyi Medical School, University of Szeged, 6720 Szeged, Hungary; sisak.anita@med.u-szeged.hu (A.S.); molnar.regina@med.u-szeged.hu (R.M.); szabo.andrea@med.u-szeged.hu (A.S.); 2 Doctoral School of Experimental and Preventive Medicine, University of Szeged, 6720 Szeged, Hungary; polanek.evelin@med.u-szeged.hu; 3 Department of Medical Physics and Informatics, Albert Szent-Györgyi Medical School, University of Szeged, 6720 Szeged, Hungary; rarosi.ferenc@med.u-szeged.hu; 4 Albert Szent-Györgyi Medical School, University of Szeged, 6725 Szeged, Hungary; armitahoseini75@gmail.com; 5 Department of Obstetrics and Gynecology, Albert Szent-Györgyi Medical School, University of Szeged, 6725 Szeged, Hungary; nemeth.gabor@med.u-szeged.hu

**Keywords:** case–control study, mental health, stress, perinatal depression

## Abstract

Perinatal depression is one of the most common mental illnesses in women. The aim of this study was to assess the association of life stressors, perceived stress, obstetric and neonatal complications, and depressive symptoms in the early postpartum period and to compare these variables in two groups of women (preterm and term deliveries). **Methods**: A case–control study was conducted among 300 women who gave birth in 2019 at the University of Szeged. Cases included women with preterm deliveries (<37 weeks, n = 100), and the controls included women with term deliveries (≥37 weeks, n = 200). Data were collected during postpartum hospital stays through a self-administered questionnaire (containing validated questionnaires: the Holmes–Rahe Life Stress Inventory, the Perceived Stress Scale (PSS-14), and the Edinburgh Postnatal Depression Scale (EPDS)) and the medical records of women and newborns. A descriptive statistical analysis and logistic regression were used to identify predictors of high EPDS scores (≥10). **Results**: Perceived stress levels were significantly higher among cases than controls (*p* < 0.001). Higher perceived stress was associated with a higher risk of depression in cases (OR: 1.31, 95% CI: 1.17–1.48, *p* < 0.001) and controls (OR: 1.33, 95% CI: 1.21–1.45, *p* < 0.001), too. Newborn complications were associated with an increased perinatal depression risk in the controls (OR: 2.48, 95% CI: 1.05–5.91; *p* = 0.039) but not in the cases (OR: 2.79, 95% CI: 0.79–9.85; *p* = 0.111). It is supposed that premature birth was stressful itself, and women with preterm babies were less sensitive to any complications occurring in their newborns compared to women with term newborns. Neither maternal age, education, nor obstetric complications predicted depressive symptoms. **Conclusions**: Our findings highlight the impact of maternal perceived stress and newborns’ health status on the risk of developing depression during the early postpartum period. These results emphasize the need for ongoing screening and follow-up measures, especially for women with higher EPDS scores.

## 1. Introduction

Preterm birth (any birth before 37 completed weeks of gestation) is a significant global health challenge, affecting approximately 4–16% of all live births worldwide [1]. It is the leading cause of death among children under the age of five [1], and those who survive preterm delivery often experience long-term health and developmental issues such as cerebral palsy, cognitive delay, and sensory impairments [2]. Preterm births can also create an emotional burden for mothers, increasing their vulnerability to psychological distress and depression in the perinatal period [3,4].

The perinatal period is a psychosocially sensitive time for women. Physical and hormonal changes, as well as normative crises such as pregnancy, childbirth, postpartum recovery, and changes in family dynamics, are all risk factors for the development of mental illness. This risk may be increased further if the pregnancy is pathological or if there are minor or major complications during childbirth [5].

One of the most common mental illnesses that threatens women in the perinatal period is perinatal depression (previously called postpartum depression) [6]. Recent reviews have stated that the term perinatal depression has replaced the previously used definition of postpartum depression in clinical practice. This includes not only depressive episodes occurring after childbirth but also those that appear during pregnancy [7].

A meta-analysis by Wang et al. with 565 studies showed that the global prevalence of perinatal depression was 17.22% in 80 countries and regions studied [8]. Perinatal depression is defined as depression in women during pregnancy and up to four weeks postpartum [9]. Symptoms of perinatal depression may include loss of interest in daily life, negative thoughts, feelings of isolation, and sadness [10]. This is not to be confused with the baby blues, which are characterized by a benign, transient, and depressed mood, usually occurring in days after birth but disappearing within 2 weeks postpartum, even without treatment [11].

Many factors may contribute to the development of perinatal depression, including normative crises (such as the transition to motherhood, physical changes, the birth of the child, and social changes) [12], feelings of guilt due to difficulties breastfeeding [13], and inadequate sleep. In the latter case, it is unclear whether this is a cause or a consequence, but it exacerbates depression and anxiety symptoms [14].

International studies have found that perinatal depression is more common in younger women, in single or widowed women, in unplanned pregnancies [15], in families with a low socioeconomic status, in women with low education levels [16], among unemployed women [17], in cases of emergency caesarean sections, if pain relief or anesthesia was not administered during birth, if a newborn’s 1 min Apgar score is less than 7 points, and in cases of preterm births [18]. A retrospective analysis by Dowse et al. (2020) further highlighted the impact of maternal mental health on birth outcomes. This study assessed depression using the EPDS (Edinburgh Postnatal Depression Scale) and analyzed its effects on neonatal outcomes such as birth weight, gestational age, Apgar scores, and NICU (neonatal intensive care unit) admission. Maternal depression was associated with lower birth weight (−63 g on average), shorter gestational age (−0.27 weeks), an increased risk of NICU admission (OR = 1.42), and longer neonatal hospital stays [5].

Previous depression has been found to increase the risk of postnatal depression threefold and of antenatal depression fourfold [19]. The consequences of untreated perinatal depression include poor sleep quality [20], breastfeeding problems (including the cessation of breastfeeding) [13], problems bonding with the newborn [21,22], weight retention, low vitality, low social functioning, low mental health, low self-esteem, inadequate anger management, lower quality of life, the development of addiction (e.g., alcohol, smoking), low social functioning, and, in more severe cases, even self-harm, suicidality, and psychosis [23].

Untreated perinatal depression has consequences not only for the woman but also for her child, e.g., the deterioration of cognitive functions, behavioral inhibition, behavioral problems, emotional maladjustment, and even the development of psychiatric disorders [23].

Based on the results of other international studies, protective factors for perinatal depression are working (but not remotely or from home), returning to work within a year after childbirth (however, long hours or psychologically stressful work are risk factors) [17], primiparity, early psychological intervention [15,24], and the intake of vitamin B6 and B12, folic acid, and omega-3 [25].

Perinatal depression is often unrecognized or underdiagnosed. Its diagnosis is complicated by the fact that symptoms of mental illness are often difficult or impossible to recognize because they can easily be mixed with the normal features of the postpartum adjustment period (e.g., hormonal changes, baby blues, adjustment to a new lifestyle, or lack of sleep due to caring for the newborn) [5]. The situation is complicated further by widespread beliefs, such as that hormonal changes during pregnancy act as a protective factor against mental illness [26], but societal expectations that childbirth should be one of the happiest periods in a woman’s life can also be harmful, which can add to the guilt in women who do not experience it this way [27].

Cultural norms also affect how perinatal mental health disorders are labeled and treated, highlighting the importance of culturally appropriate care [28]. Among migrant women, antenatal anxiety and depression have been linked to psychosocial factors such as a lack of family support and acculturation, underscoring the need for systematic, culturally sensitive screening approaches [29].

The EPDS has proven to be useful for screening for postpartum depression over the years [30,31] and may also be used to screen for antepartum depression. Knights et al. found that the EPDS questionnaire is a useful tool for screening for perinatal depression within 96 h of birth to exclude women who are not at risk. Women who scored less than 10 points on postpartum screening were 92.7% more likely to maintain this low risk level during follow-up. They found that a woman who had no psychiatric disorders before pregnancy and delivered a newborn without anomalies after 32 weeks of gestation was unlikely to score high on the EPDS. Early and late screens were well correlated, and 92% of the results remained the same or improved [32].

The aim of our study was to explore the correlation between life events during pregnancy, perceived stress, complications related to obstetrics and newborns, and the risk of perinatal depressive symptoms after delivery in women with preterm (<37 weeks of gestation) or term (≥37 weeks of gestation) [33] newborns. We hypothesized that perceived stress, obstetric complications, and newborns’ health status would be associated with a higher risk of depression in women after delivery in both groups.

## 2. Materials and Methods

### 2.1. The Study Design

A case–control study entitled “Quantifying MAternal Non-Obstetrical Risk Factors for Preterm Birth—Retrospective and Prospective Study” (the MANOR study) was performed as a collaboration between the Department of Obstetrics and Gynecology and the Department of Public Health at the University of Szeged among women delivering between March and December 2019. The data analysis presented here is part of this bigger study.

### 2.2. Participants

A total of 100 women who gave birth before the 37th week of gestation and their newborns were included in the case group, and 200 women who gave birth between the 37th and 40th week of gestation or later and their newborns were included in the control group. The use of a 1:2 case-to-control ratio is a standard and powerful design for addressing the research questions. Participation was offered to all women who delivered preterm during the study period; two term births were selected for one preterm birth, adjusted for maternal age and number of previous births, but did not take other confounder factors into account. Women completed the questionnaire during their hospital stays after delivery. The exclusion criteria in both groups were multiple pregnancies (e.g., twin pregnancies) and women younger than 18 years.

### 2.3. Measures

The data collection was based on a self-administered questionnaire, health documentation, and laboratory tests.

The questionnaire included sociodemographic-, lifestyle-, conception-, pregnancy-, delivery-, health-status-, stressor-, and stress-related questions. The variables included in the present analysis for maternal sociodemographic status are maternal age, educational level (lower and higher), type of residence, and relationship status (single or in a relationship).

The pregnancy-related questions were about complications during delivery (inadequate uterine contraction, cephalopelvic disproportion, fetal malpresentation, membrane rupture, placenta accreta, or placental abruption); these complications were transformed into a binary variable of either no complications (coded as 0) or one or more complications (coded as 1). The occurrence of diseases in the newborns (congenital anomalies, pathological jaundice, anemia, polycythemia, and other neonatal diseases, such as infections, hypoglycemia, etc.) was evaluated in the same way: no problem (coded as 0) or one or more problems (coded as 1).

The Holmes–Rahe Life Stress Inventory was used to identify stressors in women during pregnancy. The inventory included a list of 43 life events of varying severity experienced during the last year. Each event had a different “weight” (value) for stress. Higher scores have been shown to predict illness or higher stress [34].

The Hungarian validated version of the 14-item Perceived Stress Scale (PSS-14), validated by Stauder and Konkoly (2006), was used to assess perceived stress [35]. The original version of the PSS-14 was developed by Cohen et al. (1983) [36], and it is widely used in epidemiological studies. The PSS-14 focuses on one’s feelings and thoughts during the last month. Each item is rated on a 5-point scale ranging from never (0) to almost always (4). Higher total scores indicate a higher perceived stress level.

The EPDS was developed more than 30 years ago as a ten-item self-report questionnaire to facilitate screening for perinatal depression and research purposes [37,38]. It is now used in many regions of the world and has been translated into more than 60 languages. The Hungarian questionnaire was translated and validated by Töreki et al. (2013), and it is recommended to be used in clinical practice in Hungary [27]. The scale is an effective screening tool for major and minor depression at a cut-off of 9/10. Hewitt et al. reviewed the literature and supported using an EPDS cut-off score of ≥10 to detect major and minor postpartum depression [39], while Knights et al. [32] also found that women in the early postpartum period with scores <10 had a 92.7% chance of staying low-risk between 4 and 8 weeks after giving birth. Since we questioned women very early after birth, based on the mentioned literature, we chose a cut-off of 10 to better identify those who might need support. The EPDS does not diagnose mental disorders. A “high score” indicates the presence of depressive symptoms but not their duration or intensity [30]. In the present study, the EPDS score was dichotomized as 9 points or lower (depression not likely) and 10 points or higher (possible depression).

### 2.4. The Statistical Analysis

The characteristics of the study population were evaluated through descriptive statistics (numbers and percentages of responses for categorical variables and means and 95% confidence intervals for continuous variables). The independent samples *t*-test was used to compare the case and control groups with respect to the Holmes–Rahe Life Stress Inventory, the PSS-14, and the EPDS. Possible relationships between the categorical variables were analyzed using the Chi-squared test for independence. The association between the Holmes–Rahe Life Stress Inventory, PSS-14, and EPDS scores was examined using a Spearman’s correlation analysis. Univariate and backward elimination (Likelihood Ratio, LR) selected logistic regression analyses were applied to calculating the odds ratio for the high-risk EPDS category in connection with demographic, obstetrics-, and newborn-related factors; odds ratios (ORs), adjusted odds ratios (AORs), and 95% confidence intervals (95% CIs) were calculated. All analyses were conducted separately for the case and control groups. We considered a result significant if *p* < 0.05 for each statistical method. The statistical analysis was performed using the IBM SPSS Statistics 29.0 program.

### 2.5. Ethics

The study protocol was approved by the Regional and Institutional Human Medical Biological Research Ethics Committee of the University of Szeged, Hungary (approval reference number: 4419). Participation was voluntary, and written informed consent was obtained from each participant.

## 3. Results

### 3.1. The Characteristics of the Sample

Table 1 shows the sociodemographic characteristics of the study population. The age distribution of the case and control groups showed no significant difference.

Educational levels, however, differed between the groups. A higher proportion of the control group reported higher education levels (62.0%) than that in the case group (48.0%). Regarding relationship status, both groups had similar characteristics, with the majority in both groups living in a partnership (>97%). In terms of residence, more of the control group participants lived in county towns (48.2% vs. 38.0%), while a higher percentage of the case group participants lived in towns and villages.

Table 2 shows the obstetric characteristics of the women. The most frequent maternal complications were membrane rupture (premature, early, late, or high) and inadequate uterine contraction, followed by fetal malpresentation, cephalopelvic disproportion, placenta accreta, and placental abruption.

Inadequate uterine contraction occurred more frequently in the control group (36.5%) than in the cases (20.0%) (*p* = 0.004). In contrast, fetal malpresentation was nearly twice as common in the case group (14.0% vs. 6.0%) (*p* = 0.002). Membrane rupture was reported by more than half of the case group (53.0%), compared to just 26.0% of the controls (*p* < 0.001). Placenta accreta and placental abruption were exclusively reported among cases (4.0% and 3.0%).

Overall, one or more obstetric complications during delivery were reported more often among the case group (64.0%) than the control group (53.0%) (*p* = 0.070).

Table 3 shows the health problems in the newborns. In the case group, newborns were more frequently diagnosed with health problems during hospital stays. Overall, 70.0% of the infants in the case group had at least one health concern, compared to 39.0% in the control group. Pathological jaundice was more common among cases (28.0%) compared to controls (17.5%) (*p* < 0.001). The incidence of congenital anomalies, polycythemia, and anemia was similar between groups. Other neonatal diseases were infections, hypoglycemia, conjunctivitis, polycythemia, and cephalhematoma.

### 3.2. The Holmes–Rahe Stress Inventory, the EPDS, and the PSS-14

**Figure 1:**
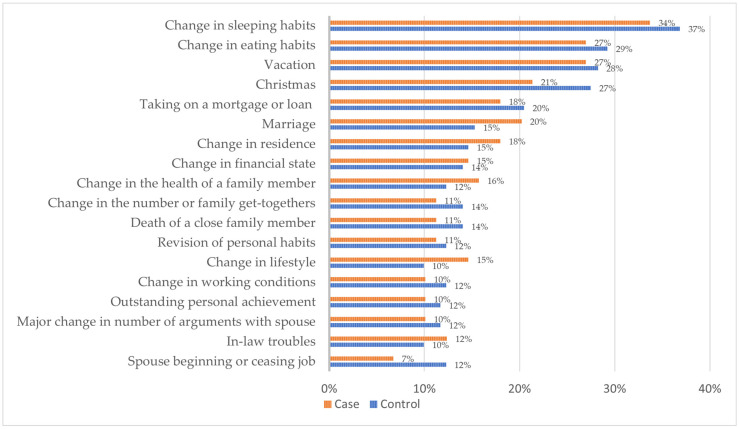
The frequency (%) of major life events during pregnancy based on the Holmes–Rahe Life Stress Inventory in the case and control groups.

The frequency of stressful life events during pregnancy, based on the Holmes–Rahe Life Stress Inventory, was similar between the case and control groups, and the statistical analysis did not reveal significant differences between them (Figure 1). The most common life events reported were changes in sleeping habits (34% of cases and 37% of controls), eating habits (27% vs. 29%), going on vacation (27% vs. 28%), and Christmas-related stress (21% vs. 27%). Moderate frequencies were also observed for major life events such as taking on a mortgage or loan (18% vs. 20%), marriage (20% vs. 15%), changes in residence (18% vs. 15%), and changes in financial state (15% vs. 14%). Family-related stressors, including health problems in a family member, the death of a loved one, and changes in family gatherings, were slightly less common but may have had strong emotional effects. Altogether, the mean score for life events did not differ significantly between the case and control groups (*p* = 0.807) (Table 4). Additionally, there was a positive correlation between cumulative life stress (Holmes–Rahe scores), perceived stress (PSS-14), and depressive symptoms (EPDS) in both groups (Table 5), suggesting that the accumulation of stressors may contribute to elevated levels of stress perception and perinatal depressive symptoms.

The EPDS scores identified 40.8% (n = 40) of the women in the case group and 25.8% (n = 50) of the women in the control group as being at risk of depression. The mean EPDS score was significantly higher in cases (mean = 9.10) than controls (mean = 6.59, *p* < 0.001)

Perceived stress, as measured using the PSS-14 scale, was significantly higher in the case group (mean = 24.47) compared to that in the controls (mean = 20.10, *p* < 0.001).

Table 5 shows the Spearman’s correlation coefficients between the Holmes–Rahe Life Stress Inventory, the PSS-14, and the EPDS in the case and control groups. Significant positive correlations were found between all three instruments in both groups. In the case group, the Holmes–Rahe Life Stress Inventory scores were fairly correlated with the PSS-14 (r = 0.298, *p* < 0.01) and poorly correlated with the EPDS (r = 0.235, *p* < 0.05). However, a moderate correlation was observed between the PSS-14 and the EPDS (r = 0.701, *p* < 0.01). Similarly, in the control group, the Holmes–Rahe Life Stress Inventory scores correlated fairly with the PSS-14 scores (r = 0.350, *p* < 0.01) and poorly with the EPDS scores (r = 0.242, *p* < 0.01), while the PSS-14 scores showed moderate correlation with the EPDS scores (r = 0.724, *p* < 0.01). These results indicate a consistent and significant relationship between perceived stress and depressive symptoms in both groups, with stress from life events also playing a role.

Table 6 summarizes the results of the univariable logistic regression models assessing the odds of high EPDS scores, indicating an elevated risk of postpartum depression, across both study groups. Among the cases, higher PSS-14 scores were significantly associated with higher EPDS scores (OR = 1.29, 95% CI: 1.16–1.44, *p* < 0.001). A similar association was observed in the control group (OR = 1.32, 95% CI: 1.21–1.44, *p* < 0.001). Neither obstetric complications, newborn health problems, nor age had statistical significance in predicting the EPDS risk in either the case group or the control group.

Table 7 shows the results of the final step of the backward elimination selected logistic regression models in the case and control groups. Age and obstetric complications were eliminated from both models. Ultimately, the likelihood of a high risk of depression was significantly increased by increased perceived stress in both the case (OR: 1.31, 95% CI: 1.17–1.48, *p* < 0.001) and control groups (OR: 1.33, 95% CI: 1.21–1.45, *p* < 0.001). Additionally, the presence of disease in newborns was also associated with a higher risk of depression in the control group (OR: 2.48, 95% CI: 1.05–5.91; *p* = 0.039), while this association was not significant in the case group (OR: 2.79, 95% CI: 0.79–9.85; *p* = 0.111). The Nagelkerke R-squares (0.512 in cases and 0.491 in controls) indicate that both models explain about 50% of the variability in the outcomes.

## 4. Discussion

This study assessed the correlation between stressful life events, perceived stress, complications related to obstetrics and newborns, and the risk of perinatal depressive symptoms among women with preterm or term newborns. We found that the prevalence of high EPDS scores (≥10) was 40.8% in the case (preterm) and 25.8% in the control (term) groups. Another finding of our study was that perceived stress may be a significant predictor of perinatal depressive symptoms (an EPDS score ≥ 10), regardless of whether the delivery is preterm or term.

Several studies have emphasized the relationship between stress and depression during pregnancy and the postpartum period. Papapetrou et al. conducted a prospective cohort study among 178 pregnant women using the EPDS to assess depressive symptoms during pregnancy [40]. Their findings revealed that 26.9% had significant depressive symptoms (an EPDS score ≥ 10), a number which is slightly higher than the value found in our control group (25.8%).

Studies have already revealed the psychosocial, biological, and socioeconomic risk factors that can contribute to perinatal depression [12,13,14,15,16,17,19,23]. Our results further highlight the impact of maternal perceived stress and newborn health status on the risk of developing maternal perinatal depression during the early postpartum period.

The number and type of life events during pregnancy were similar in both the case and control groups according to the Holmes–Rahe Life Stress Inventory, while women who gave birth preterm reported higher perceived stress levels as measured using the PSS-14. This could mean that how women feel about stress may be more subjective than actual stressful life events themselves. However, we found a correlation between perceived stress and EPDS scores in both groups, suggesting that perceived stress may contribute to the onset of perinatal depression. These results correspond to findings from the international literature [41,42,43,44]. There are additional explanations for this observation. For example, women who are more sensitive to stress may also be more vulnerable to developing depression, or a poor stress coping mechanism may result in depression later on [45]. Furthermore, considering the biological and/or psychological mechanisms of stress and depressive symptoms, previous research has proposed possible pathways that may explain the observed association between stress and depressive symptoms. Changes in the hypothalamic–pituitary–adrenal (HPA) axis, including elevated cortisol levels; a reduced coping capacity; and increased cognitive reactivity may contribute to emotional vulnerability in the postpartum period due to perceived stress [46,47].

Our study did not show a significant association between higher EPDS scores and the women’s age; complications during pregnancy and delivery; and health problems in newborns in either group, though in previous studies these factors were found to be significant risk factors for perinatal depression [15,16,18]. A meta-analysis by Cárdenas et al. found that preterm births, emergency cesarean sections, and severe pain were associated with increased depressive symptoms in the postpartum period [3]. These studies included larger and multicenter samples, applied broader definitions of complications, and measured depressive symptoms in a later period postpartum. Our findings might be different due to the single-site study design; the fact that the screening was conducted shortly after delivery; or the Hungarian antenatal supportive care system, in which obstetricians, midwives, and health visitors work in close collaboration with the expectant mother. All of these factors may have reduced the psychological impact of complications.

Neonatal complications were more common in the preterm group, yet a significant association with depressive symptoms was only observed in the control group. A possible explanation could be that complications after term births are less expected, leading to more emotional distress compared to that in the mothers of preterm infants. This interpretation is also partly supported by previous studies, like the findings of Dadi et al., who reported a relationship between perinatal depression and adverse infant outcomes in their systematic review [48].

To strengthen our results, it is essential to recognize that the observed disparities in obstetric and neonatal complications arise from the inherent clinical differences between the case and control groups. This interpretation reinforces our observation that despite these expected medical differences, perceived stress (PSS-14) proved to be the most reliable predictor of depressive symptoms within both groups.

The results emphasize the need for ongoing perinatal depression and stress screening using validated instruments such as the EPDS and the PSS-14. Follow-up measures and personalized interventions are especially important for women with higher perinatal EPDS scores and higher perceived stress levels and whose children have experienced neonatal complications.

### 4.1. Clinical and Academic Implications

Routine screening for perinatal depression using the EPDS during postpartum hospital stays is favorable, as it provides an opportunity to reach a larger number of women and to identify those at a higher risk of depression, which allows healthcare professionals to facilitate early interventions or offer follow-up and support.

The EPDS is a quick screening tool that can be administered and evaluated without the need for trained personnel, making it effective for identifying women with a higher risk of perinatal depression.

Our findings further confirmed that elevated maternal stress might be an early warning sign before the onset of depressive symptoms. Perinatal depression can occur even after uncomplicated deliveries; however, closer monitoring may be required for mothers whose newborns have neonatal complications.

### 4.2. Limitations

As the data collection was retrospective and based on a self-administered questionnaire, inaccuracies might be present in the recall of the women’s memories.

As the EPDS was assessed based on life events that had occurred in the last few days of pregnancy and the first few days postpartum, we could not separate cases of baby blues from PPD cases, and the EPDS was probably not a suitable screening tool.

Finally, this study was conducted at a single regional medical center in Hungary, and its findings may not be widely generalizable. Regional health policies, clinical protocols, and patient populations might differ in other regions and countries. Therefore, multicenter studies would be necessary to validate and extend our results.

## 5. Conclusions

This study reinforces that perceived stress plays an important role in predicting perinatal depressive symptoms independently from the outcomes of pregnancy, while its novelty lies in identifying neonatal complications as risk factors, which may increase the risk of depressive symptoms in mothers with term newborns.

Even a temporary disorder in their newborns can be considered an unexpected, additional trauma for mothers who have had problem-free pregnancies and deliveries. These mothers are less prepared for newborn-related complications than, for example, those who have spent more time in the pregnancy pathology department before delivery. Within the framework of personalized postpartum care, in addition to the integration of perceived stress and perinatal depression screening into routine clinical practice, special attention should also be paid to mothers whose term newborns have health problems.

As our research was a retrospective study, a longitudinal design would be recommended to validate our findings. Furthermore, a nationwide multicenter or multicultural study would be beneficial to allow for more general conclusions.

## Figures and Tables

**Table 1:** Demographic characteristics of case and control groups.

Sociodemographic Characteristics		Case (n = 100) n (%)	Control (n = 200) n (%)
	−29	29 (29.0)	61 (30.5)
Age-group (years)	30–34	35 (35.0)	67 (33.5)
	35–	36 (36.0)	72 (36.0)
	lower	47 (47.0)	75 (37.5)
Educational level	higher	48 (48.0)	124 (62.0)
	missing data	5 (5.0)	1 (0.5)
	single	3 (3.0)	4 (2.0)
Relationship status	in a partnership	97 (97.0)	193 (98.0)
	missing data	0 (0.0)	3 (1.5)
	county town	38 (38.0)	96 (48.2)
Residence	town	35 (35.0)	54 (27.1)
	village	26 (26.0)	49 (24.6)
	missing data	1 (1.0)	1 (0.5)

**Table 2:** Characteristics of delivery in case and control groups.

Characteristics		Case n (%)	Control n (%)
Inadequate uterine contraction	Yes	20 (20.0)	73 (36.5)
	No	80 (80.0)	127 (63.5)
Cephalopelvic disproportion	Yes	3 (3.0)	19 (9.5)
	No	97 (97.0)	181 (90.5)
Fetal malpresentation	Yes	14 (14.0)	12 (6.0)
	No	86 (86.0)	188 (94.0)
Membrane rupture	Yes	53 (53.0)	52 (26.0)
	No	47 (47.0)	248 (74.0)
Placenta accreta	Yes	4 (4.0)	0 (0.0)
	No	96 (96.0)	200 (100.0)
Placental abruption	Yes	3 (3.0)	0 (0.0)
	No	97 (97.0)	200 (100.0)
Summary of obstetric complications	One or more complications	64 (64.0)	106 (53.0)
	No complication	36 (36.0)	94 (47.0)

**Table 3:** Characteristics of newborns in case and control groups.

Characteristics		Case n (%)	Control n (%)
Congenital anomalies	Yes	12 (12.0)	21 (10.5)
	No	88 (88.0)	179 (89.5)
Pathological jaundice	Yes	28 (28.0)	35 (17.5)
	No	72 (72.0)	165 (82.5)
Anemia	Yes	2 (2.0)	4 (2.0)
	No	98 (98.0)	196 (98.0)
Polycythemia	Yes	2 (2.0)	6 (3.0)
	No	98 (98.0)	194 (97.0)
Other neonatal diseases	Yes	52 (52.0)	42 (21.0)
	No	48 (48.0)	158 (79.0)
Summary of health problems in newborns	One or more problems	70 (70.0)	78 (39.0)
	No problem	30 (30.0)	122 (61.0)

**Table 4:** The mean scores in the Holmes–Rahe Life Stress Inventory, PSS-14, and EPDS in the case and control groups.

Variables	Case		Control		*p*-Value ^1^
	Mean	SD	Mean	SD	
Holmes–Rahe	142.81	81.94	140.01	90.23	0.807
PSS-14	24.47	7.70	20.10	7.20	<0.001
EPDS	9.10	6.43	6.59	5.02	<0.001

^1^ Independent samples *t*-test; SD: standard deviation; PSS-14: 14-item Perceived Stress Scale; EPDS: Edinburgh Postnatal Depression Scale.

**Table 5:** The Spearman’s correlation analysis between life-event-related stress (Holmes–Rahe Life Stress Inventory), perceived stress (PSS-14), and postnatal depressive symptoms (EPDS) in the case and control groups.

Variables	Case		Control	
	Holmes–Rahe	PSS-14	Holmes–Rahe	PSS-14
PSS-14	0.298 **	1	0.350 **	1
EPDS	0.235 *	0.701 **	0.242 **	0.724 **

* Correlation is significant at the 0.05 level. ** Correlation is significant at the 0.01 level. PSS-14: 14-item Perceived Stress Scale; EPDS: Edinburgh Postnatal Depression Scale.

**Table 6:** The odds of a high risk of depression according to the EPDS scores—the results of univariate binary logistic regression in the case and control groups.

Variables	Case			Control		
	OR	95% CI	*p*-Value	OR	95% CI	*p*-Value
Age group (years)						
≤29	ref.			ref.		
30–34	0.91	0.32–2.60	0.858	0.46	0.21–1.03	0.060
35-	2.26	0.82–6.22	0.074	0.49	0.22–1.07	0.074
Obstetric complications						
yes	1.36	0.59–3.18	0.471	0.92	0.48–1.75	0.791
no	ref.			ref.		
Diseases in newborns						
yes	1.95	0.788–4.88	0.151	1.51	0.78–2.89	0.217
no	ref.			ref.		
PSS-14 (continuous)	1.29	1.16–1.44	<0.001	1.32	1.21–1.44	<0.001

OR: odds ratio; 95% CI: 95% confidence interval; PSS-14: 14-item Perceived Stress Scale.

**Table 7:** The odds of a high risk of depression according to EPDS scores—the last step of backward (LR) logistic regression in cases and controls.

Variables	Case			Control		
	OR	95% CI	*p*-Value	OR	95% CI	*p*-Value
Diseases in newborns						
yes	2.79	0.79–9.85	0.111	2.48	1.05–5.91	0.039
no	ref.			ref.		
PSS-14 (continuous)	1.31	1.17–1.48	<0.001	1.33	1.21–1.45	<0.001
Nagelkerke R square		0.512			0.491	

OR: odds ratio; 95% CI: 95% confidence interval; PSS-14: 14-item Perceived Stress Scale. LR: Likelihood Ratio. Variable(s) removed at step 2: obstetric complications; variable(s) removed at step 3: age group—year of survey: 2019.

## Data Availability

The dataset used and analyzed during the current study is available from the corresponding authors on reasonable request.

## Abbreviations

The following abbreviations are used in this manuscript:

AOR	adjusted odds ratio
CI	confidence interval
EPDS	Edinburgh Postnatal Depression Scale
LR	Likelihood Ratio
NICU	neonatal intensive care unit
OR	odds ratio
PSS	Perceived Stress Scale
